# Modifying Factors for Concussion Incidence and Severity in the 2013-2017 National Hockey League Seasons

**DOI:** 10.7759/cureus.3530

**Published:** 2018-10-31

**Authors:** Ryan Adams, Adam Y Li, Jennifer B Dai, Syed Haider, George K Lau, Kevin P Cheung, Alexander F Post, Alex Gometz, Tanvir F Choudhri

**Affiliations:** 1 Neurosurgery, The Icahn School of Medicine at Mount Sinai, New York, USA; 2 Miscellaneous, Touro College of Medicine, Bronx, USA; 3 Neurosurgery, Columbia University, New York, USA; 4 Physical Medicine and Rehabilitation, Concussion Management of New York, New York, USA

**Keywords:** concussion rate, style of play, national hockey league, home/away, win/loss, length of game, points scored, time of season, player ice position and concussion

## Abstract

Introduction

In the past few years, there has been a rising interest in both the prevalence and the short- and long-term consequences of concussions. While the main focus of concussion-based research revolves around the National Football League (NFL), attention is now shifting to other high contact leagues like the National Hockey League (NHL), where there is constant player-to-player contact as well as collisions with the perimeter boards. While the body of evidence surrounding injury and concussion rates in the NHL has substantially grown in size over the previous few years, there is still a void pertaining to the in-game effects that could modulate concussion incidence. Our study takes a novel approach to evaluate several “style of play” factors such as home/away perspective, win/loss outcome, points scored, real time length of game, time of season, and player position in modulating concussion rates.

Methods

Data on concussion incidence for the 2013-2017 National Hockey League seasons was collected utilizing FOX Sports injury tracker. Only injuries specifically diagnosed as concussions during regular and postseason games were utilized in our data set. A Google search on the reported injury was performed in order to correlate the concussion to the correct game in which the player sustained it. NHL season schedules were acquired through the online source “Hockey Reference.” There were a total of 5281 games when considering the regular and postseason games between the 2013-2017 seasons. Concussions sustained during team practices and preseason contests were not accounted for in our data set to control for inconsistent reporting. Our data set does not account for the current 2017-2018 NHL expansion with the addition of a Las Vegas team to the league.

Results

Statistical analysis of several "Style of Play" factors such as home/away perspective, win/loss outcome, points scored, real time length of game and time of season produced non-significant results pertaining to modulating concussion rate during the 2013-2017 NHL seasons. When evaluating on-ice position we noted offensive players combined to have the highest rate of concussion. Forwards (left wing (LW), right wing (RW)) demonstrated similar concussion rates, while goalies encountered the lowest concussion rate.

Conclusion

The results of our analysis demonstrated non-significance for home/away effects, win/loss results, average points scored, real time length of game, and time of season on influencing concussion rates. We noted offensive players combined to have the highest rate of concussions, while goalies encountered the least. The key limitation in our data set is the lack of reliable and publicly available data surrounding concussion incidence in the National Hockey League. Due to this drawback, our data set should be considered as an under-reported representation of the total amount of concussions spanning the 2013-2017 seasons.

## Introduction

In the past few years, there has been a rising interest in both the prevalence and the short- and long-term consequences of concussions. While the main focus of concussion-based research revolves around the National Football League (NFL), attention is now shifting to other high contact leagues like the National Hockey League (NHL), where there is constant player-to-player contact as well as collisions with the perimeter boards. It has been shown that concussions occur at all skill and age levels in ice hockey and have been reported to account for 2%–14% of all hockey injuries and 15%–30% of all hockey head injuries [1].

This awareness and increasing body of literature surrounding traumatic brain injury has led to several advances in concussion injury prevention. For instance, more research has begun on head-impact biomechanics, rules have been changed to limit injury exposure, equipment viability has been explored, and nutritional supplementation has been added to many players' diets [2]. Further investigation into concussions in the NHL has demonstrated that the reported concussion rate in the NHL during the last five years is more than triple that of the previous decade. Bigger, faster players, new equipment, and harder boards and glass have all theoretically increased the risk of concussion in the NHL in recent years [3].

A report published in the Canadian Medical Association Journal examined physician reports from the 1997-2004 NHL seasons. They noted a total of 559 concussions during regular-season games [4]. This implied a concussion rate of 5.8 for every 100 players, or an estimated 1.8 concussions per 1,000 player-hours [4]. A separate study by Wennberg et al. demonstrated that there has been a gradual increase in the average number of games missed per concussion during the 1997-2008 seasons. This study evaluated time lost from play data that was available for 310 concussions during the first five seasons of the study period and for 288 concussions during the last five seasons. The mean number of missed games per concussion during the last five seasons was significantly greater than during the first five seasons (12.9 ± 17.8 vs. 8.3 ± 13.1) [5].

While the body of evidence surrounding injury and concussion rates in the NHL has substantially grown in size over the previous few years, there is still a void pertaining to the in-game effects that could modulate concussion incidence. One recent study evaluated the effects of in-game fatigue on concussion rates. Their data suggest that in-game fatigue is an important factor when considering concussions. They noted a player's average ice time per game was a significant predictor of concussion, while total ice time for the season was not. There was also no significant difference observed in the number of games played in the season between concussed and non-concussed players [6].

Our study takes a novel approach to evaluate several “style of play” factors such as home/away perspective, win/loss outcome, points scored, real time length of game, time of season and player position in modulating concussion rates. We hope the results of this analysis shed light on the mechanisms by which in-game factors affect concussion incidence in the National Hockey League.

## Materials and methods

Data collection

One caveat to our data collection was the striking lack of publicly available data pertaining to the concussions sustained by each NHL team. We noted an under-reporting of concussions in our data set on a season-to-season basis compared to similar papers evaluating concussions in the NHL [7].

Data on concussion incidence for the 2013-2017 National Hockey League seasons was collected utilizing FOX Sports injury tracker [7]. Only injuries specifically diagnosed as concussions during regular and postseason games were utilized in our data set. A Google search on the reported injury was performed in order to correlate the concussion to the correct game in which the player sustained it. NHL season schedules were acquired through the online source “Hockey Reference.” There were a total of 5281 games when considering the regular and postseason games between the 2013-2017 seasons. Total postseason games varied season to season based upon how quickly teams advanced through the seven game series to the Stanley Cup Final. Concussions sustained during team practices and preseason contests were not accounted for in our data set to control for inconsistent reporting [7].

Our data set does not account for the current 2017-2018 NHL expansion with the addition of a Las Vegas team to the league.

Concussion rate across major contact sports

National Football League (NFL)

The concussion rate was acquired through a previously unpublished work produced by our study team. PBS Frontline was utilized for the source data. [Unpublished Original Article: Haider S, Choudhri T. The effects of environmental factors on concussion risk and incidence in National Football League from 2012 – 2015; 2017].

National Hockey League (NHL)

The concussion rate was acquired through a previously published work produced by our study team. FOX Sports injury tracker was utilized for source data. We acknowledge a limitation in this data set due to a lack of publicly available data pertaining to concussion injuries and consider it to be an under-reported representation of the total number of concussions that occurred between the 2013-2017 NHL seasons [7].

Australian Football League (AFL)

The concussion rate was acquired through a previously unpublished work produced by our study team. The data was acquired from the weekly injury list published by the Australian Football League. [Unpublished Original Article: Adams R, Lau G, Dai J, et al. Evaluation of concussion incidence and modulating factors in the 2013-2017 Australian Football League; 2018].

Definition 

Style of Play Factors

Home/away, win/loss, points scored, length of game, time of season, and positional analysis.

Concussion Rate

(Total number of concussions under a specific condition) / (Total number of games inclusive of that specific condition).

Real Time Length of Game

Real length of game is composed of three 20-minute periods and two 17 minute intermissions. We also considered TV time outs at minutes 10, 8, and 6 incorporated into the time. These TV time outs can span around 45 seconds to two full minutes.

Time of Season

In order to evaluate the concussion rate during specific times of the season, we broke down the NHL season into three segments. The beginning of the season was defined as October – December, the middle of the season was denoted as January – March, and the end of the season was defined as April – June.

On-ice Position

Center (C), defense (D), left wing (LW), right wing (RW), and goalie (G) are the on-ice positions.

Statistical analysis

The data was analyzed utilizing GraphPad Prism 6 (GraphPad, La Jolla, CA). In addition to descriptive statistics, Fisher's exact tests, Welch’s two tailed t-tests, and correlation tests were used. An alpha level < 0.05 was considered significant for all tests.

## Results

Our data set was compiled from NHL teams participating in the 2013-2017 seasons. Team demographics such as city and arena were acquired from our online sources (Table 1). One important caveat for our analysis was the omission of the recent Las Vegas expansion team whose first season was the end of 2017 through 2018 [7]​​​​​​​.

**Table 1:** NHL Teams Participating in the 2013-2017 Seasons Arena presented is representative of where the team played for the 2013-2017 seasons.

Team	Abbreviation	City	Arena
Anaheim Ducks	ANA	Anaheim, CA	Honda Center
Arizona Coyotes	ARI	Glendale, AZ	Gila River Arena
Boston Bruins	BOS	Boston, MA	TD Garden
Buffalo Sabers	BUF	Buffalo, NY	KeyBank Center
Calgary Flames	CGY	Calgary, AB	Scotiabank Saddledome
Carolina Hurricanes	CAR	Raleigh, NC	PNC Arena
Chicago Blackhawks	CHI	Chicago, IL	United Center
Colorado Avalanche	COL	Denver, CO	Pepsi Center
Columbus Blue Jackets	CBJ	Columbus, OH	Nationwide Arena
Dallas Stars	DAL	Dallas, TX	American Airlines Center
Detroit Red Wings	DET	Detroit, MI	Joe Lewis Arena
Edmonton Oilers	EDM	Edmonton, AB	Rogers Place
Florida Panthers	FLA	Sunrise, FL	BB&T Center
Los Angeles Kings	LAK	Los Angeles, CA	Staples Center
Minnesota Wild	MIN	St. Paul, MN	Xcel Energy Center
Montreal Canadians	MTL	Montreal, QC	Bell Centre
Nashville Predators	NSH	Nashville, TN	Bridgestone Arena
New Jersey Devils	NJD	Newark, NJ	Prudential Center
New York Islanders	NYI	New York, NY	Barclays Center
New York Rangers	NYR	New York, NY	Madison Square Garden
Ottawa Senators	OTT	Ottawa, ON	Canadian Tire Centre
Philadelphia Flyers	PHI	Philadelphia, PA	Wells Fargo Center
Pittsburgh Penguins	PIT	Pittsburgh, PA	PPG Paints Arena
San Jose Sharks	SJS	San Jose, CA	SAP Center
St. Louis Blues	STL	St. Louis, MO	Scottrade Center
Tampa Bay Lightning	TBL	Tampa, FL	Amalie Arena
Toronto Maple Leafs	TOR	Toronto, ON	Air Canada Centre
Vancouver Canucks	VAN	Vancouver, BC	Rogers Arena
Washington Capitals	WSH	Washington, D.C.	Verizon Center
Winnipeg Jets	WPG	Winnipeg, MB	Bell MTS Place

We broke down our data set into individual seasons in order to gauge the distribution of all 133 concussions across the 2013-2017 NHL seasons (Table 2). Concussion incidence was also broken down from the home and away team perspectives. A few games in our data set registered multiple concussions in a single game, which resulted in concussions appearing in 2% of all games between the 2013-2017 NHL seasons [7].

**Table 2:** Demographic Analysis of Concussion Incidence Demographic analysis of concussion incidence broken down on a season-to-season basis, home vs. away perspective, and total games with a concussion.

Demographics Data of Included Team Games (N = 5281)		
Variable	n	%
2013-2014 season	36	27
2014-2015 season	27	20
2015-2016 season	24	18
2016-2017 season	46	35
Total Number of Concussions	133	-
Home Team Concussions	71	53
Away Team Concussions	62	47
Total Games with Concussion	125	2
Total Games without Concussion	5156	98

**Figure 1:**
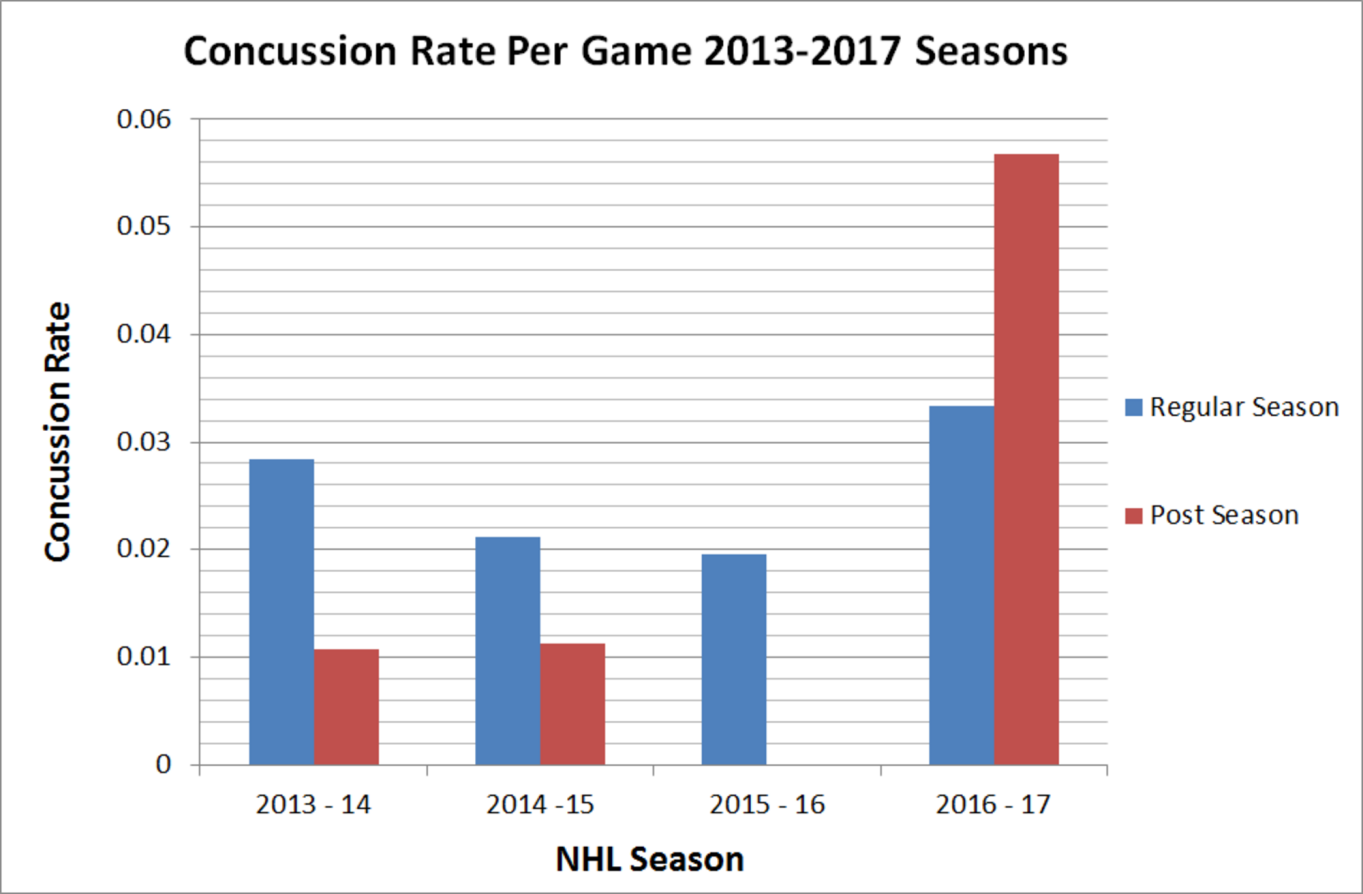
Concussion Rate Per Game Concussion rate per game analyzed in both the regular season and postseason for the 2013-2017 NHL seasons. No concussions were documented in the 2015-2016 postseason utilizing the FOX Sports injury tracker.

The concussion rate per game was tabulated for each individual regular and postseason game between the 2013-2017 NHL seasons (Figure 1). Of note was the absence of any reported concussions during the 2015-2016 postseason. This could be due to the lack of publicly available data surrounding concussion incidence from the NHL [7].

**Figure 2:**
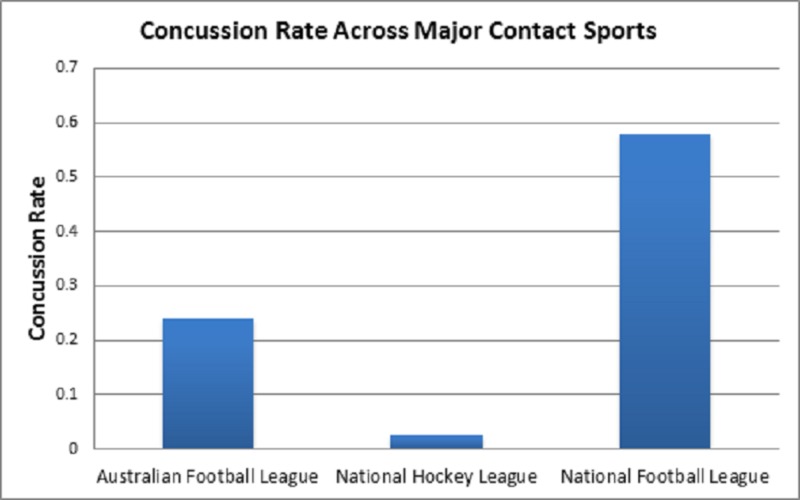
Concussion Rate Across Major Contact Sports Calculated concussion rate per game comparison between the National Hockey League (NFL), the National Football League (NFL), and Australian Football League (AFL). NHL, NFL and AFL concussion rate was determined by our research group in previously published works [8-11]. No statistical tests were run on this observational finding.

Evaluating the concussion rate across major contact sports was a cornerstone of our research group’s analysis (Figure 2). Prior works composed by our research group discerned the average concussion rate in both the NHL and NFL [7]. We noted an average concussion rate of 0.025 in the NHL and 0.58 in the NFL. Analysis indicated a concussion rate of 0.24 in the AFL. Due to the variations in rules, concussion management, and play style across these sports, we provided this graph of our group’s findings for observational purposes in lieu of testing for statistical significance.

**Figure 3:**
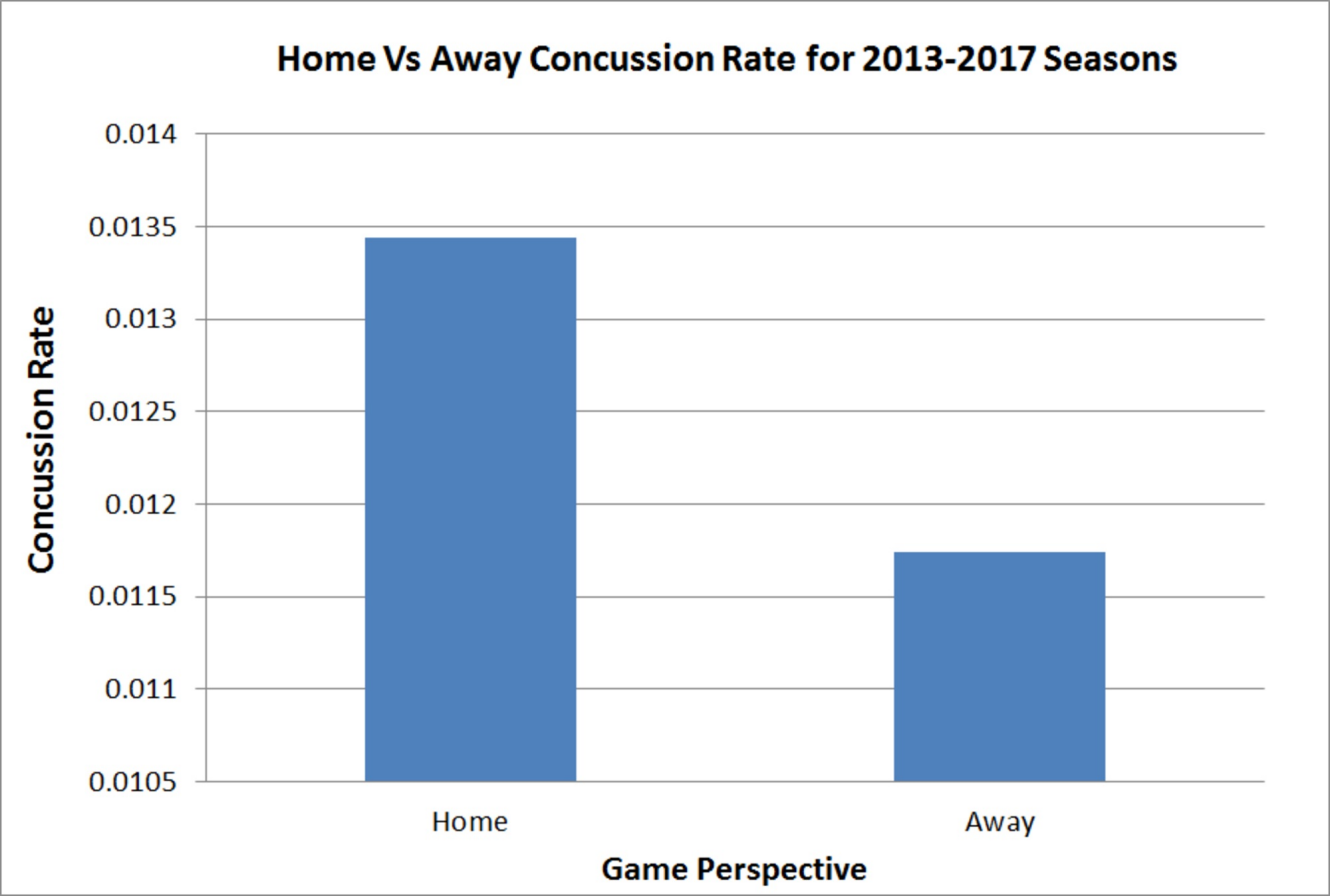
Home vs. Away Concussion Rate for 2013-2017 NHL Seasons Analysis of the concussion rate/game from the home vs. away team perspective during the 2013-2017 NHL seasons. Results of this analysis demonstrated non-significance (p = 0.4584).

There are a multitude of benefits towards playing from the home advantage during a competitive sporting event. Our group wanted to evaluate whether the home/away perspective contributed towards an increase in concussion risk for players (Figure 3). We noted no significant difference when comparing the concussion rate between the home and away teams for the combined 2013-2017 NHL seasons (p = 0.4584).

**Figure 4:**
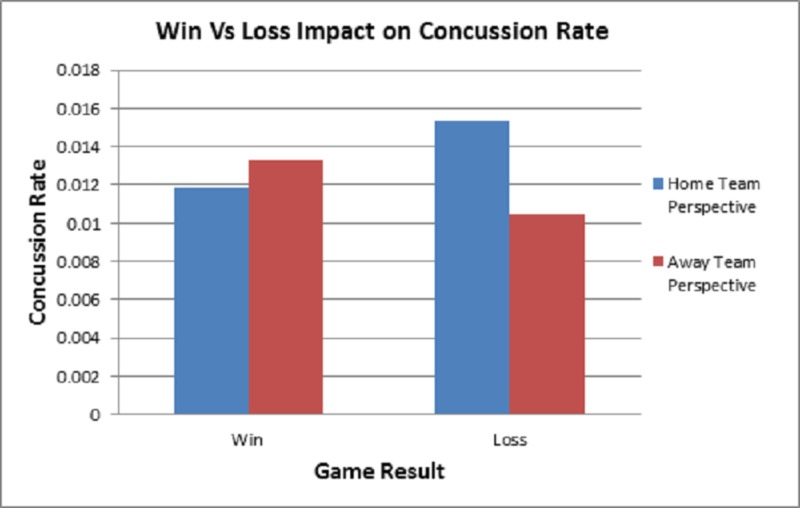
Win vs. Loss Impact on Concussion Rate Analysis of the concussion rate/game from a win vs. loss perspective between the 2013 and 2017 NHL seasons. Results of this analysis demonstrated non-significant findings.

One facet of the style of play measures which we hypothesized to have an impact on concussion rate was the win/loss game outcome (Figure 4). The results of this analysis demonstrated non-significance when comparing home-loss vs away-win (p = 0.4161), home-win vs away-loss (p = 0.8153), home-win vs home-loss (p = 0.3149), and away-win vs away-loss (p = 0.6757).

**Figure 5:**
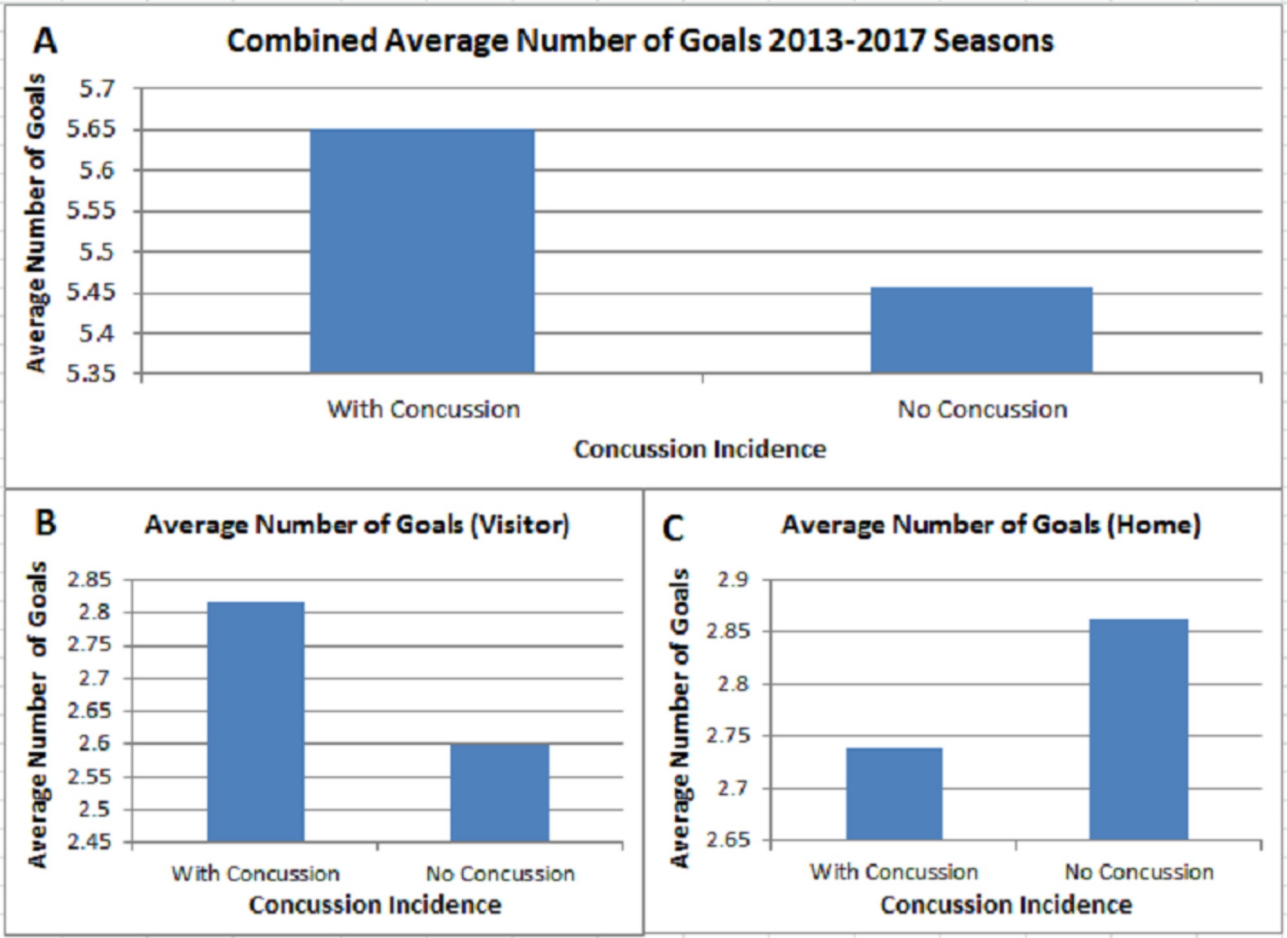
Average Points Scored Analysis Analysis of average goals scored in games with or without concussion during the 2013-2017 NHL seasons. Results of the analysis indicated a non-significant difference (p = 0.3472)

Analysis of the average number of goals scored during games with and without a concussion was performed in order to evaluate its implication in modulating concussion rate (Figure 5A). We combined the 2013-2017 seasons and noted no significance in the average goals scored between games with and without a concussion (p = 0.3472). We also ran the statistics for both the home and visiting teams independently to see if there were any changes to their respective concussion rate (Figures 5B, 5C). We noted no significance in the findings as well.

**Figure 6:**
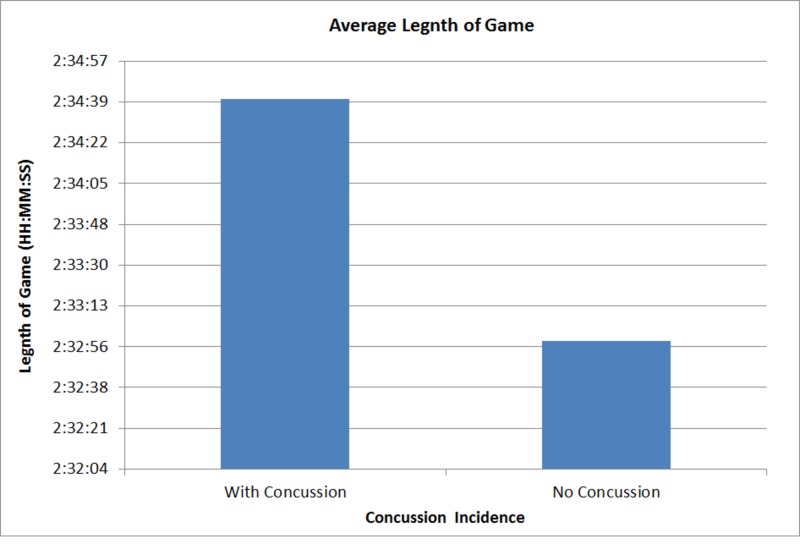
Real Time Length of Game Real time average length of game with and without a concussion during the 2013-2017 NHL seasons. Real length of game is comprised of three 20 minute periods and two 17 minute intermissions. We also considered TV time outs at minutes 10, 8 and 6 incorporated into the time. These TV time outs can span around 45 seconds to 2 full minutes. Final result was shown to be non-significant (p = 0.2069).

We were interested in determining if the real time length of game had any implications on the concussion rate over the course of the season. Game length with a concussion was compared to those games in which no concussion occurred. The result demonstrated non-significance for the effect of real time length of game influencing concussion rate (p = 0.2069) (Figure 6).

**Figure 7:**
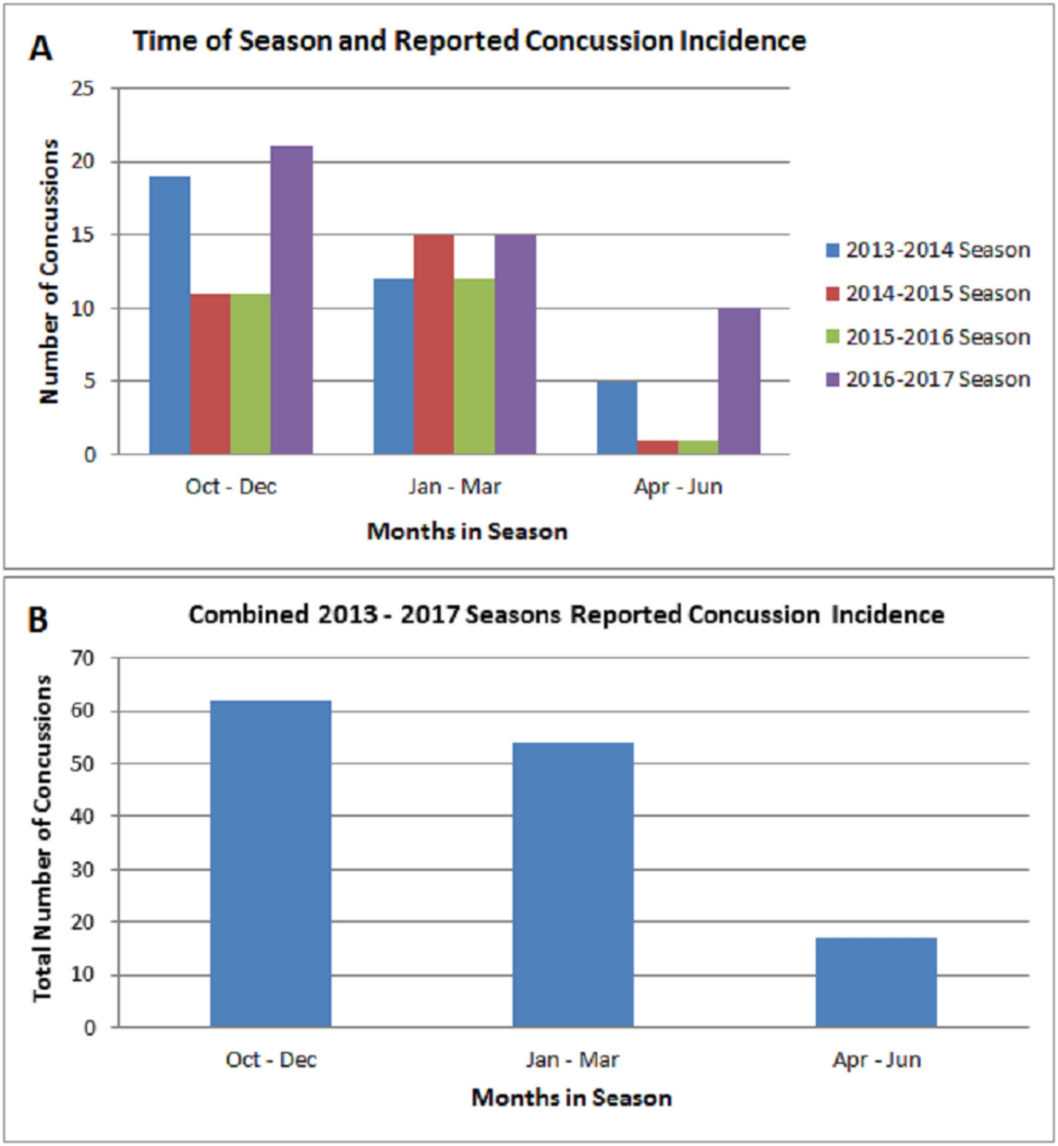
Time of Season Analysis A) Time of season and reported concussion analysis for each individual season between 2013 and 2017. B) We noted no significance in the findings when comparing the first third of the season (Oct-Dec) to the final third of the season (Apr-Jun) (p = 0.7490). No significant difference was noted for the first third vs. the middle third (p = 0.4468).

Another component of our style of play evaluation was to determine if the time of season had any implications on concussion incidence. We broke down each season into thirds in order to adequately capture the beginning, middle, and end of the season (Figure 7A). Data analysis demonstrated no significance when comparing the beginning of the season to the middle (p = 0.4468) or the beginning of the season to the end of the season (p = 0.7490). However we noticed a trend indicating fewer concussions reported in the final three months of the season when compared to other time points (Figure 7B).

**Figure 8:**
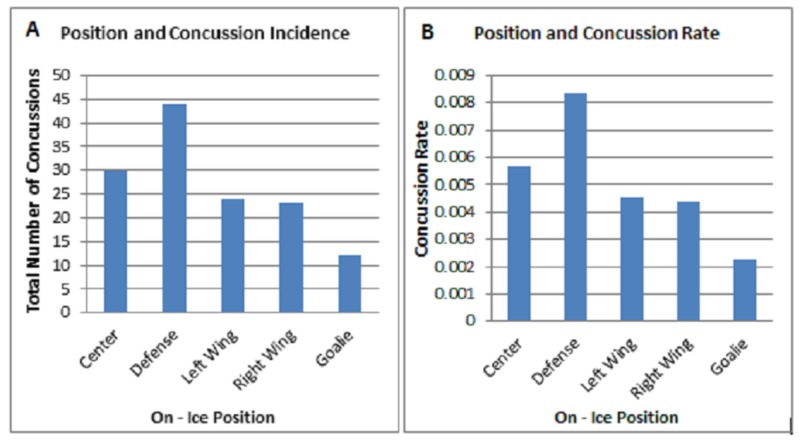
On-ice Position Analysis A) Evaluation of concussion incidence for on-ice positions during the 2013-2017 NHL seasons. B) Total number of concussions at each position was divided by total number of games in our data set (5281 games) to attain rate for the 2013-2017 seasons.

The final component of our style of play analysis involved evaluating concussion risk per on-ice player position (Figure 8A). We were interested in seeing if certain players were more at risk for sustaining a concussion because of their specific role on the ice. We identified player position for all 133 concussions in our data set and noted offensive players (C , LW, and RW) combined to have the highest rate of concussions, while goaltenders encountered the least. The concussion rate amongst left and right wingers was roughly similar with centers having a slightly higher rate (Figure 8B).

## Discussion

Game characteristics have been reported to affect both the incidence and severity of concussions in sports. For example, several studies in the National Football League (NFL) have reported differences in concussion incidence due to a variety of factors including running vs. passing plays, different play strategies such as the West Coast offense scheme, strength of schedule, days of rest between games, timing of games during the season, game location, game outcome, and player position [8, 9].

In the NHL, epidemiological data on concussions and all injuries is more limited [1]. Hutchison et al. reviewed all NHL injuries during 2006-2010 seasons and found that impacts causing concussions were primarily at the concussed player’s head (68%) due to contact with the opponent’s shoulders, elbows, or gloves. In addition, the hitting player was taller than the injured player in 52% of cases and heavier in 65% of cases [10, 11]. Two studies have looked into the effects of player position and have found that offensive players have disproportionately more concussions than other positions. While offensive players compose half the players on the ice rink, they have been reported to suffer 64%-65% of concussions [5, 11]. Fatigued players have also been reported to suffer more concussions, as playing for 15 minutes or more was significantly associated with higher concussion incidence [1, 6]. Players' goals, assists, points, plus-minus, penalty minutes, blocked shots, hits, giveaways, and time on the ice were not statistically different before and after concussion injury [12].

Our study looked further into the effects of game characteristics on concussion incidence but did not find any significant outcomes. It can be noted that the most recent season, 2016-2017, had the highest concussion rate out of the four seasons studied, and home teams had more concussions than away teams, while not significant [7]. This disagrees with a previous study by our group on concussion incidence in the NFL, which showed that away teams had a significantly higher concussion incidence than home teams [Unpublished Original Article: Haider S; 2017]. In addition, our study demonstrates a trend that offensive players (C, LW, and RW) were more likely to suffer a concussion than other positions as 58% of concussions were by offensive players compared to defensemen and goalies. NHL benches typically contain four groups of three offensive players, consisting 12 out of 19 players on a bench. Our study and previous studies analyzing concussion incidence within player positions agree with a conclusion of a higher incidence for offensive players as opposed to defensemen and goalies [5, 10].

Time of season also had no major effect on concussion incidence unlike other sports, which is likely due to a controlled playing environment inside of a stadium instead of open stadiums in other sports. Although fewer concussions occurred April-June, the NHL playoffs begin in April, so the decrease in concussion rate is likely due to the decrease in games played. In addition, while there were slight differences in concussion rates due to game outcome, goals scored, and length of game, none of these differences are truly significant or meaningful.

It is interesting to note the divergence between the concussion incidence per game between the NFL, AFL, and NHL, with the biggest difference between the NFL and NHL (Figure 2). While we believe this difference to be attributed to our limited data sets and publicly available data, past studies have shown that ice hockey has a higher rate of concussion incidence compared to football [13, 14]. Importantly, these past studies looked at college sports, used concussion incidence/1000 athlete exposures instead of concussion incidence/game, and reported relatively low concussion rates in ice hockey, (0.27 and 0.41)/1000 athlete exposures. If our study quantified results using athlete exposures, the contrast between sports would still be present, as we report a 6.3 concussions/1000 athlete exposure rate in the NFL and a 0.67 concussions/1000 athlete exposure rate in the NHL. This low NHL concussion rate may be a reason why we were not able to find any significant results in our study.

Other studies also noted the lower than expected rate of concussion incidence in the NHL, as well as a supposed declining concussion rate over time [1]. The highest reported mean concussion rate was 97 concussions/NHL season between 1997-2002, and later studies report decreasing concussion rates over time down to 33.3/NHL season in our study [1]. Authors from other studies concluded that this may be due to many reasons including under-reporting of symptoms to avoid missing games, more conservative management by medical staff, higher thresholds for diagnosis of concussions, and increased neuropsychological testing results before allowing return to play [4, 15]. If true, these claims would indicate both under-reporting of primary concussion injury as well as prevention of repeat concussions by ensuring complete recovery before return to play [1]. This is important, as Donaldson et al. indicated that incidents of unspecified upper body injuries resulting from trauma to the head with symptoms described as “concussion-like” frequently occur on the NHL, and concussion incidence between 2009-2011 would nearly double if “concussion-like” injuries were included [16]. Therefore, changes to protocol regarding primary reporting of concussion injury in the NHL may lead to both increased concussion incidence and increased player safety.

The key limitation in our data set is the lack of reliable and publicly available data surrounding concussion incidence in the National Hockey League. The ideal data set would be comprised of medical diagnosis from the team doctors detailing injury date and diagnosis, complemented by neuro-psychometric data pertaining to return to play clearance. Due to this drawback, our data set should be considered as an under-reported representation of the total amount of concussions spanning the 2013-2017 seasons. Thus, our data should be understood as only a sampling of the total concussions during the examined NHL seasons.

## Conclusions

Our study sought to evaluate the effects of several “style of play” factors on concussion incidence between the 2013 and 2017 National Hockey League seasons. The results of our analysis demonstrated non-significance for home/away effects, win/loss results, average points scored, real time length of game, and time of season on influencing concussion rates. We also evaluated on-ice position in order to determine which players were most susceptible to in-game concussions. We noted defensemen had the highest rate of concussions, while goaltenders encountered the least.
